# Common genomic variants associated with breast cancer predict the risk of second primary breast cancer diagnosis

**DOI:** 10.1186/1897-4287-10-S2-A28

**Published:** 2012-04-12

**Authors:** S Sawyer, J McKinley, G Mitchell, G Chenevix-Trench, M Harris, G Lindeman, PA James

**Affiliations:** 1 Peter MacCallum Cancer Institute, Australia; 2 Queensland Institute of Medical Research, Australia; 3 Monash Medical Centre, Australia; 4 Royal Melbourne Hospital, Australia; 5 Victorian Clinical Genetics Service, Australia

## Background

A diagnosis of a second primary breast cancer (‘bilateral breast cancer’) occurs in between 2-11% of women affected by breast cancer in the general population, but is increased in frequency in women with a family history of breast cancer. We investigated whether the common genomic variants described in breast cancer GWAS studies were determinants of the risk of bilateral breast cancer and compared the effect to that of family history.

## Methods

The Victorian FCC Translational Breast Cancer Cohort consists of the index cases of 1374 families with a high risk family history of breast or ovarian cancer. The cohort has been extensively characterised, including detailed personal and family history and has undergone genetic testing for both high penetrance genes and low penetrance variants associated with breast cancer. 1143 women in the cohort have had at least one breast cancer and 208 women have had multiple primary breast cancers. For this study genotypic data from 22 common genomic variants (SNPs) previously identified in breast cancer GWAS were analysed for each index case and compared to the same set of genotypes from an Australian population based control group (n=895, recruited from the electoral role for the AOCS). A Polygenic Risk Score (PRS) was calculated using a multiplicative model as the sum of the log odds ratios associated with each allele. Unilateral and bilateral cases were compared on the basis of both the measured polygenic risk and family history parameters.

## Results

768 women affected by breast cancer were identified in whom BRCA1 or 2 mutation was excluded by sequencing and MLPA. The cohort was followed up an average of 8.8 years since their diagnosis of breast cancer with 135 (18%) women having a diagnosis of a second primary breast cancer. The average Polygenic Risk Score in the group was 1.30 (Log OR) but was significantly increased in the women with a history of bilateral versus unilateral breast cancer (1.40 vs 1.28 p=0.018), with no difference between the groups in follow up (or time to cancer diagnosis) (7.3 vs 7.8 years, p=0.50) or age of first diagnosis (44.8 vs 45.5, p=0.47). Measures of the family history including the number of breast cancers in first degree or any relatives, family history of bilateral breast cancer, male breast cancer or ovarian cancer were also examined but showed no significant differences between women with unilateral or bilateral breast cancer.

To examine the clinical significance of the difference in PRS the cohort was divided into high, intermediate and low polygenic risk groups. The OR for bilateral breast cancer between the high and low risk groups was 1.9 (95% CI 1.07-3.49, p=0.027) and survival analysis showed a significantly increased rate of second primary breast cancer in the high versus low polygenic risk group (cumulative hazard to 20 years of 0.39 vs 0.24).

## Conclusion

Analysis of polygenic risk, determined by common genomic variants, has the potential to stratify women affected by familial breast cancer in relation to their risk of second primary breast cancer, independent of measures of family history.

